# Regulation of Actg1 and Gsta2 is possible mechanism by which capsaicin alleviates apoptosis in cell model of 6-OHDA-induced Parkinson's disease

**DOI:** 10.1042/BSR20191796

**Published:** 2020-06-25

**Authors:** Jiahui Liu, Hong Liu, Zhenxiang Zhao, Jianfeng Wang, Dandan Guo, Yiming Liu

**Affiliations:** 1 Department of Neurology, Qilu Hospital of Shandong University, Jinan, Shandong Province 250012, China; 2 Central Hospital of Baotou, Baotou, Inner Mongolia autonomous Region 014040, China; 3 Department of Neurology, People's Hospital of Liaocheng Affiliated to Taishan Medical College, Liaocheng, Shandong Province 252000, China

**Keywords:** Actg1, apoptosis, Gsta2, neuroprotection, Parkinson's disease

## Abstract

The present study aimed to identify the gene expression changes conferred by capsaicin in the cell model of 6-OHDA-induced Parkinson's disease, to disclose the molecular mechanism of action of capsaicin. We used capsaicin-treated and paraffin-embedded wax blocks containing substantia nigra tissue from 6-OHDA-induced Parkinson's disease rats to analyze transcriptional changes using Affymetrix GeneChip Whole Transcript Expression Arrays. A total of 108 genes were differentially expressed in response to capsaicin treatment, and seven of these genes were selected for further analysis: Olr724, COX1, Gsta2, Rab5a, Potef, Actg1, and Acadsb, of which Actg1 (actin gamma 1) was down-regulated and Gsta2 (Glutathione S-transferase alpha 2) was up-regulated. We successfully overexpressed Actg1 and Gsta2 *in vitro*. CCK-8 detection and flow cytometry demonstrated that overexpression of Actg1 and Gsta2 increased apoptosis in the 6-OHDA-induced Parkinson's disease cell model. The imbalance between Actg1 and Gsta2 may be one of the mechanisms of cell damage in Parkinson's disease (PD). Capsaicin can protect the cells and reduce the apoptosis rate by regulating Actg1 and Gsta2.

## Introduction

Parkinson's disease (PD) is a progressive neurodegenerative disease characterized by selective death and degeneration of dopaminergic neurons in the substantia nigra. One major objective of PD research is to explore potential disease-modifying drugs that slow or stop the underlying neurodegenerative process [1,2]. In previous research, our lab found that capsaicin could protect against oxidative insults and alleviates behavioral deficits in rats with 6-OHDA-induced PD via activation of transient receptor potential vanilloid subfamily member 1 (TRPV1) [3], but we have not shown the molecular mechanism of action of capsaicin.

Capsaicin is an irritant ingredient found in red pepper, which can cause body temperature to rise, increase blood flow, increase energy consumption and reduce oxidative stress. Capsaicin is an effective antioxidant, even if it is eaten in a short period of time, it also helps to reduce low-density lipoprotein [4,5]. Capsaicin can also be locally applied to the treatment of peripheral neuropathy and can regulate the damage of rat liver mitochondrial membrane (RLM) caused by gamma radiation. Capsaicin can effectively inhibit radiation-induced biochemical changes, including lipid peroxidation and protein oxidation. It can also significantly prevent radiation-induced loss of antioxidant enzyme and important endogenous antioxidant glutathione activity, and can be used as antioxidant and radiation protection agent in physiological systems [6]. Capsaicin can regulate the redox state of red blood cells. As an antioxidant, capsaicin can increase the level of glutathione and may help prevent age-related diseases [7]. The neurotoxic dose of capsaicin can change the sensitivity of neutrophils to bacterial stimulation, and the effect is related to the level of neuropeptides [8].

Capsaicin is an alkaloid found primarily in the fruit of the Capsicum genus [9], and is the classical agonist of a nonselective cation channel, TRPV1 [10,11]. Traditionally, TRPV1 has been considered to be involved in a broad range of diseases [12]. Recently, more and more studies have found that TRPV1 is not only highly expressed in sensory neurons but also present in various regions of the whole brain. Moreover, experiments have suggested that TRPV1 can significantly reduce deficits in motor and cognitive functions [13]. The preliminary experiments of our lab showed that capsaicin is mainly involved in rescuing nigral neuron survival by inhibiting oxidative stress on the ipsilateral side, protecting against DA neuron loss. Therefore, TRPV1 might be an effective neuroprotective target for PD [14]. Here, we demonstrated that capsaicin can protect against 6-OHDA-induced Parkinson's disease and reduce the apoptosis rate by regulating Actg1 and Gsta2 in a cell model.

Actins are a family of highly conserved cytoskeletal proteins that play fundamental roles in nearly all aspects of eukaryotic cell biology [15]. The ability of a cell to divide, move, endocytose, generate contractile force and maintain shape is reliant upon functional actin-based structures. The Actg1 gene provides instructions for making a protein called gamma (γ)-actin, which is part of the actin protein family. Proteins in this family are organized into a network of fibers called the actin cytoskeleton, which makes up the structural framework inside cells. These proteins play important roles in determining cell shape and controlling cell movement (motility). Recently, one study demonstrated that embryonic development is delayed and cell growth and survival are impaired in Actg1 null mice. Actg1-deficient cells exhibit growth impairment and reduced cell viability [15]. Another study showed that systemic dysregulation of cellular cytoskeletal genes is involved in HBx-induced hepatocarcinogenesis [16].

Glutathione S-transferase 2 (Gsta2) is a member of the glutathione S-transferase (GST) superfamily, which encodes multifunctional enzymes important in the detoxification of electrophilic molecules, including carcinogens, mutagens and several therapeutic drugs, by conjugation with glutathione. Gsta2 is one of the functional antioxidant response elements (AREs), which play a role in defense against oxidative stress [17]. Recently, one study demonstrated that up-regulating Gsta may serve as a compensatory mechanism against elevated oxidative stress, which accompanies obesity. On the other hand, down-regulating Gsta can impair the defense against oxidative stress [18]. Here, our experiments demonstrated that overexpression of Actg1 (actin gamma 1) and Gsta2 increased apoptosis in a cell model of 6-OHDA-induced Parkinson's disease. We further demonstrated that capsaicin may protect the cells and reduce the apoptosis rate by regulating Actg1 and Gsta2.

## Materials and methods

### Cell culture

Human neuroblastoma SH-SY5Y cells were provided by the Otolaryngology Institute of Qilu Hospital of Shandong University and maintained at 5% CO_2_ at 37°C in MEM:Ham's F12 (1:1) supplemented with 10% fetal bovine serum, penicillin (100 µnits/ml), and streptomycin (100 μg/ml). Cells were subcultured weekly. All experiments were performed between passages 25–35 and 90% cell confluence. The cells were seeded into multiwell plates. The cell culture medium was replaced every 3 days [19].

### Cell model establishment

Well-growing SH-SY5Y cell suspensions were seeded into 24-well plates, and then 6-OHDA at the final concentration of 12.5–100 µmol/l diluted with 0.2% vitamin C solution was added for 0–48 h [20–22]. Then, the CCK-8 and Annexin V-FITC flow cytometry kit were used to detect the cell survival rate. According to the experimental results and literature report [23–25], we finally established the SH-SY5Y cell model, selecting 50 μmol/l 6-OHDA for 24 h as the model treatment [25–27].

### Affymetrix genechip analysis

In our previous experiment: Capsaicin was treated intraperitoneally for the 6-OHDA induced PD rats and the locomotor activity and abnormal involuntary movements were found alleviated. Besides, we found that brain oxidative insults were investigated relieved. And the immunostaining of substantia nigra further suggested that capsaicin might protect against dopaminergic neuronal loss [3]. Then we analyzed the transcriptional changes in paraffin-embedded wax blocks containing substantia nigra tissue from capsaicin-treated rats with 6-OHDA-induced Parkinson's disease using Affymetrix GeneChip Whole Transcript Expression Arrays. Microarray hybridization, data collection and analysis were performed at Oebiotech Biotechnology Corporation according to Agilent protocols. Detailed procedures are provided in the Supplementary Methods. A total of 108 genes were differentially expressed in response to capsaicin treatment, and seven of these genes were selected for further analysis: Olr724, COX1, Gsta2, Rab5a, Potef, Actg1 and Acadsb.

### Construction of overexpression vectors for the seven genes

#### ‘Rat–human’ gene matching

The seven genes that may be closely related to PD identified in the above experiments were compared and analyzed by using the GenBank database. The most homologous and functional human genes were identified for each gene, the relevant gene sequences were downloaded from the NCBI database, and primers were designed with DNA STAR software.

#### Construction of expression vectors

Total RNA was extracted from PD model cells using the RNAprep Pure Cell-Bacteria Kit (Affymetrix Rat Clariom™ D slug and version 13.1, Agilent Technologies software), and cDNA synthesis was performed with the TIANScript RT Kit. The high fidelity DNA polymerase Pfu DNA polymerase was used to amplify the PCR, and the target gene fragments were obtained. Then, the PCR products were purified by the TIANquick Maxi Purification Kit, and the PCR products of each target gene and the eukaryotic expression vector pcDNA3.1-myc/His (−) A were digested by restriction endonuclease Xho I and BamH I. The double enzyme-digested products were recovered and purified with the TIANgel Maxi Purification Kit. The target gene fragment was connected to the skeleton fragment of the expression vector by T4 DNA ligase to form a circular recombinant plasmid. The ligation product was transformed into *E. coli* and screened with an LB/Agar plate containing ampicillin. The positive clones were identified by PCR. A small amount of plasmid was extracted by the TIANprep Rapid Mini Plasmid Kit. An LB/Agar plate containing ampicillin was used to screen the positive clones, which were identified by bacterial fluid PCR, and the plasmid was extracted by the TIANprep Rapid Mini Plasmid Kit.

#### Detection of the RNA expression level

Relative Q-PCR quantification was carried out before and after transfection of the seven overexpression vectors, and the changes in the RNA level were observed. Through extraction of total RNA from PD model cells by RNAprep Pure Cell-Bacteria Kit directly and reverse transcription of cDNA, we detected the RNA expression level of the abovementioned seven genes by SYBR Green relative quantitative Q-PCR. We used 2-△△Ct method for data processing.

#### Detection of the protein expression level

The seven genes were cloned into the same eukaryotic expression vector pcDNA3.1, and the eukaryotic expression plasmids were constructed. These plasmids were transfected into PD model cells. We have carried out three independent experiments, each of which has been repeated three times. Then WB (western blot) detection and IF (immunofluorescence) detection with His-tag and HA-tag, respectively, were used. Only the expression of the proteins Gsta2 and Actg1 could be detected.

### Apoptosis assay

#### Analysis of apoptosis by flow cytometry

Cell apoptosis was measured using an Annexin V-FITC/PI Apoptosis Detection kit. Briefly, the seven overexpression plasmids were transfected into SH-SY5Y cells individually. After 4 h of transfection, 6-OHDA at a final concentration of 50 µmol/l was added, and cells were incubated at 37°C and 5% CO_2_ for 24 h. All the cells were collected by flow cytometry, washed with sterile PBS two times, and stained with anti-Annexin V-FITC/PI. Apoptosis was analyzed by a flow analyzer.

#### Analysis of apoptosis by the CCK-8 detection kit

Cell apoptosis was measured using the CCK-8 apoptosis detection kit. Briefly, seven overexpression plasmids were transfected into SH-SY5Y cells individually. After 4 h of transfection, 6-OHDA was added at a final concentration of 50 μmol/l, and cells were incubated at 37°C and 5% CO_2_ for 24 h. The medium was replaced with new cell culture medium, and the CCK-8 reagent (50 μl/well) was added, followed by incubation in a cell incubator for 2 h. The supernatant was then transferred to a 96-well plate with 100 μl/pore, and each sample divided into four separate wells. The cell proliferation curve was immediately drawn by measuring the absorption value at 450 nm with a multifunctional enzyme marker. Cell culture medium (400 μl/well) was added to a 24-well plate, and cell imaging was performed with an inverted microscope.

### Statistical analysis

All statistical analyses were carried out using GraphPad Prism 6.0 software. The data are presented as the mean ± SEM. *P* values of less than 0.05 were considered statistically significant. All statistical analyses were performed with the SPSS 16.0 software. In the figures, asterisks indicate the degree of significance when compared with the controls, such as *P*<0.01.

## Results

### Differentially expressed genes of capsaicin-treated cells

**Figure 1:**
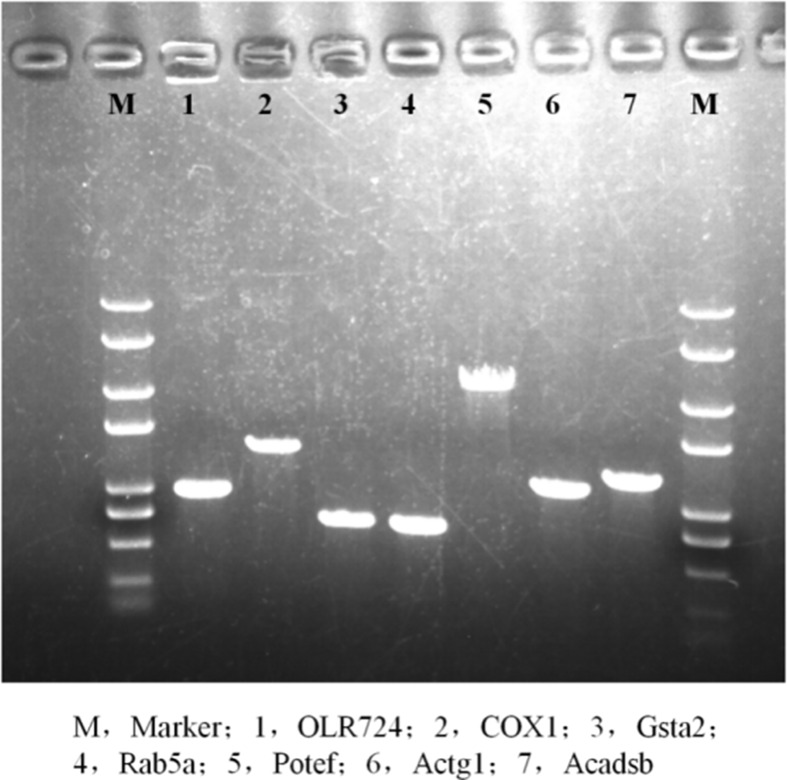
Amplified fragments of seven target genes were subjected to agarose gel electrophoresis The results showed that seven target genes were successfully amplified.

In previous studies, we showed that capsaicin protects against oxidative insults and alleviates behavioral deficits in rats with 6-OHDA-induced Parkinson's disease. We used the Affymetrix GeneChip Whole Transcript Expression Arrays to analyze the capsaicin-treated and paraffin-embedded wax blocks containing substantia nigra tissue from rats with 6-OHDA-induced Parkinson's disease. Comparing the control group with that treated with capsaicin revealed 108 differentially expressed genes (DEGs). GO analysis showed that 26 genes were up-regulated and 45 genes were down-regulated. Pathway analysis showed that 15 genes were up-regulated and 34 genes were down-regulated. Combined with the above two analyses, we found that there was overlap among the following seven genes and related pathways. Therefore, we selected these seven genes as target genes for further analysis: Olr724, COX1, Gsta2, Rab5a, Potef, Actg1 and Acadsb. Figure 1 shows the selection process of the seven differentially expressed genes. Table 1 shows the seven genes and their related pathways.

**Table 1:** The list of seven related genes and the pathways involved

Gene	Official full name	Fold changes	Function description
Olr724	Olfactory receptor 724	2.38	Olfactory transduction, etc.
COX1	Mitochondrially encoded cytochrome c oxidase I	2.32	Parkinson's disease; oxidative phosphorylation, etc.
Gsta2	Glutathione S-transferase alpha 2	2.05	Glutathione metabolism, etc.
Rab5a	RAB5A, member RAS oncogene family	−2.02	Regulation of long-term neuronal synaptic plasticity; phagosome, etc.
Potef	POTE ankyrin domain family member F	−5.18	Phagosome, etc.
Actg1	Actin gamma 1	−3.18	Phagosome, etc.
Acadsb	Acyl-CoA dehydrogenase short/branched chain	−2.47	Fatty acid degradation and metabolism, etc.

### Successful construction of the overexpression vector

**Figure 2:**
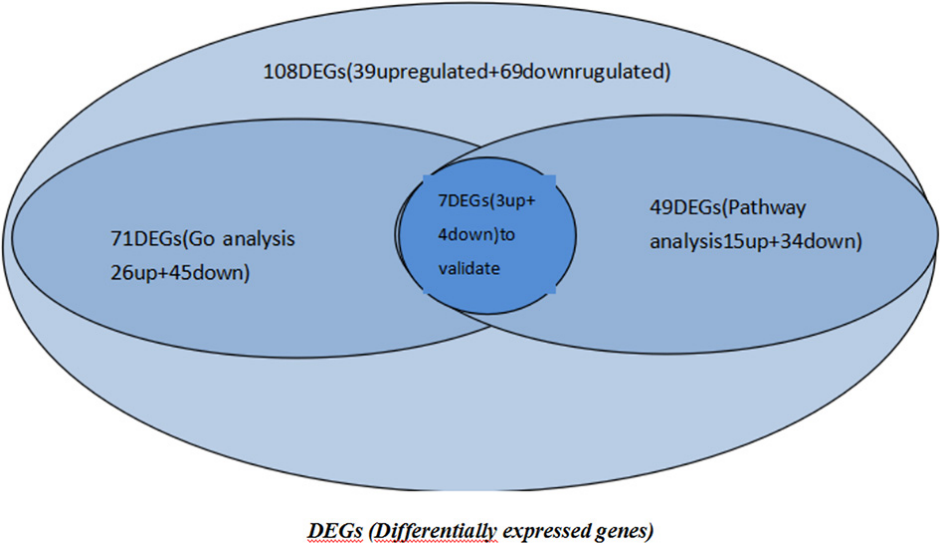
A Venn diagram of the selected genes We select seven genes as our target genes from the involved 108 genes.

**Figure 3:**
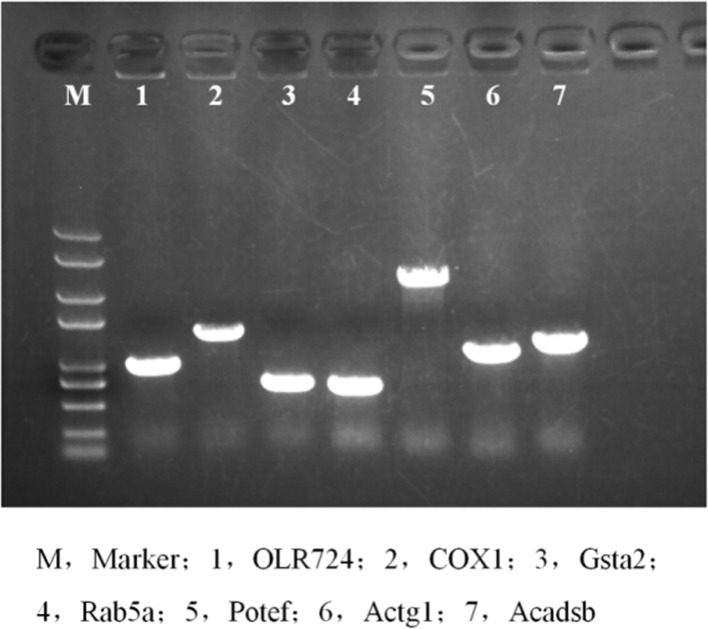
Identification of bacterial solution by PCR The results showed that seven target genes were successfully cloned into the plasmids.

**Figure 4:**
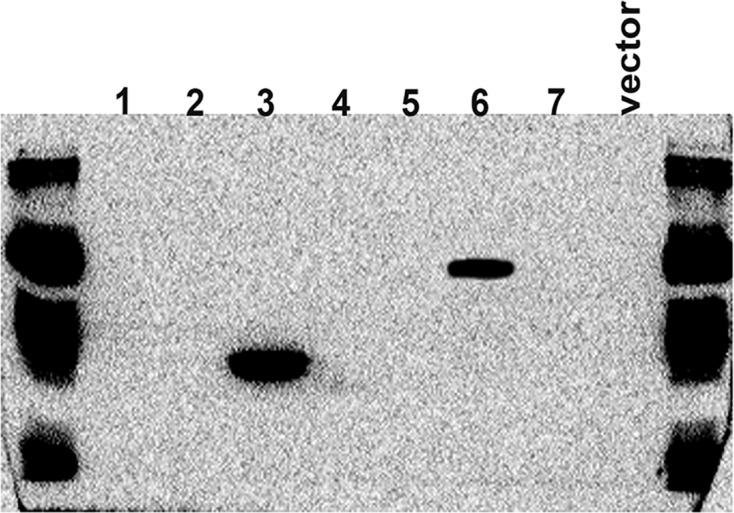
Protein quantification in SH-SY5Y cells transfected with seven target gene overexpression vectors by WB with His-tagged proteins The results showed that only two target genes (Gsta2 and Actg1) were highly expressed in the cells

**Figure 5:**
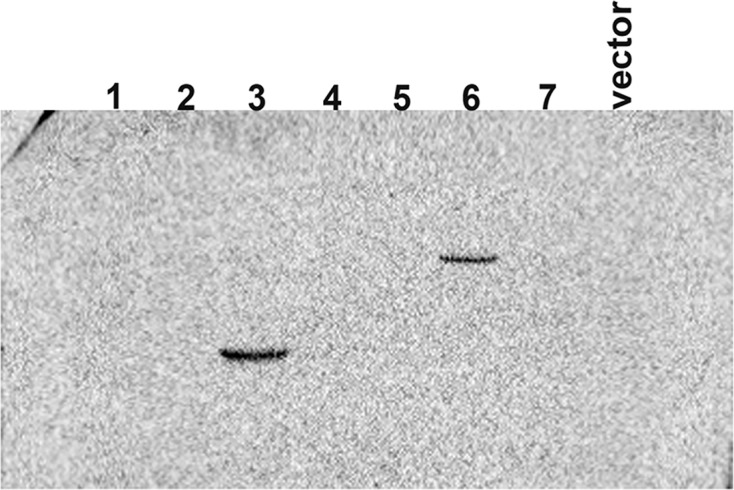
Protein quantification in SH-SY5Y cells transfected with seven target gene overexpression vectors by WB with HA-tagged proteins The results showed that only two target genes (Gsta2 and Actg1) were highly expressed in the cells

**Figure 6:**
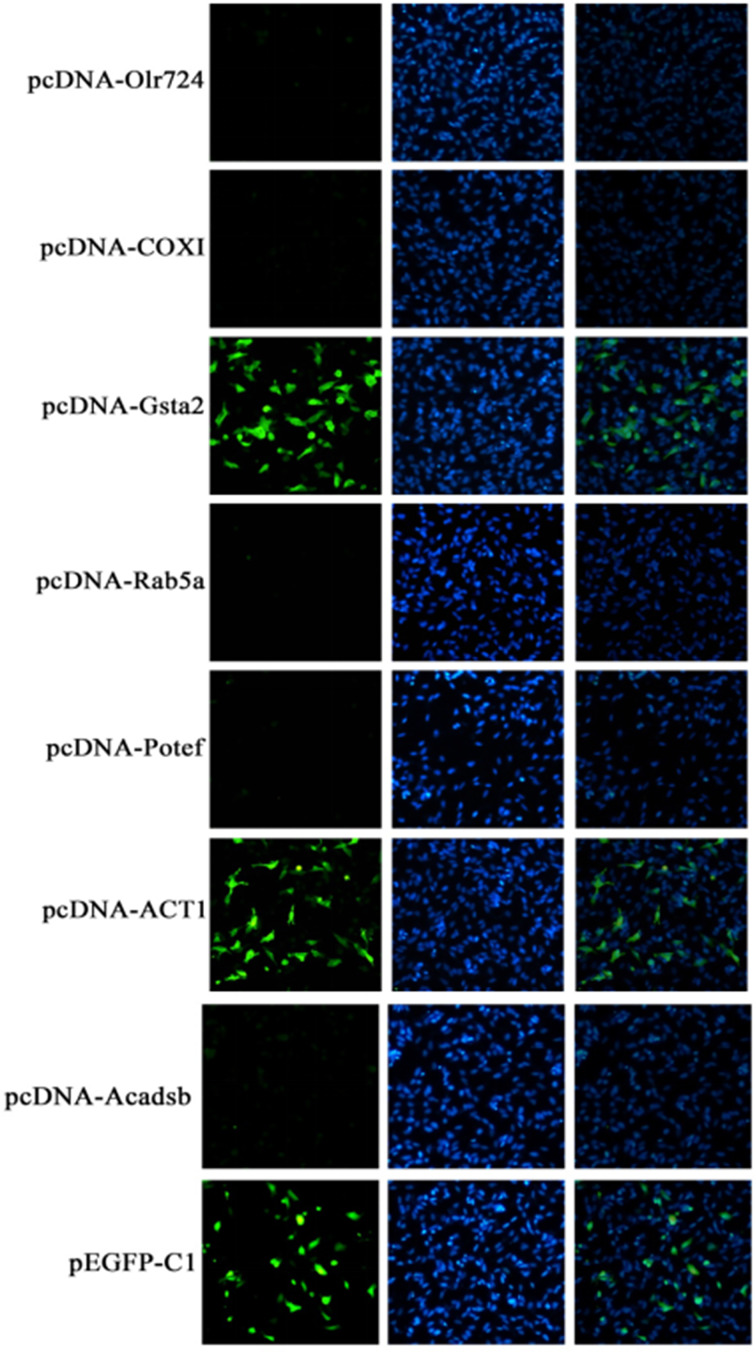
IF quantification of protein in SH-SY5Y cells transfected with seven target gene overexpression vectors The results showed that only two target genes (Gsta2 and Actg1) were highly expressed in the cells. 1, pcDNA-Olr724; 2, pcDNA-COXI; 3, pcDNA-Gsta2; 4, pcDNA-Rab5a; 5, pcDNA-Potef; 6, pcDNA-Actg1; 7, pcDNA-Acadsb; 8, pEGFP-C1

We constructed overexpression vectors of the seven target genes and then transfected them into SH-SY5Y cells. Figure 2 shows target gene amplification results. Figure 3 is the identification result of bacterial liquid PCR, indicating that the overexpression vector was constructed successfully. Furthermore, we transfected the seven overexpression vectors into SH-SY5Y cells and the protein expression level was detected by WB (Figures 4 and 5) and IF (Figure 6). Only two of the seven genes, Gsta2 and Actg1, were detected. Gsta2 and Actg1 were successfully overexpressed in SH-SY5Y cells.

### Apoptosis detection results

**Figure 7:**
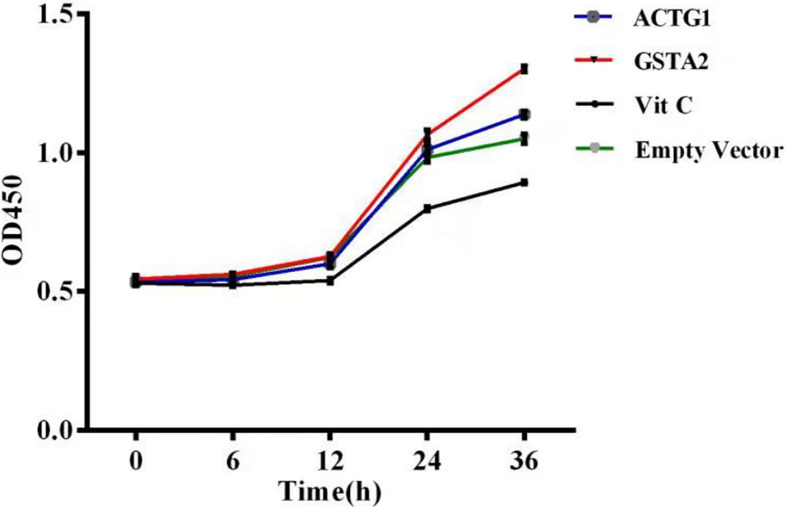
The apoptosis results determined by the CCK-8 apoptosis kit

**Figure 8:**
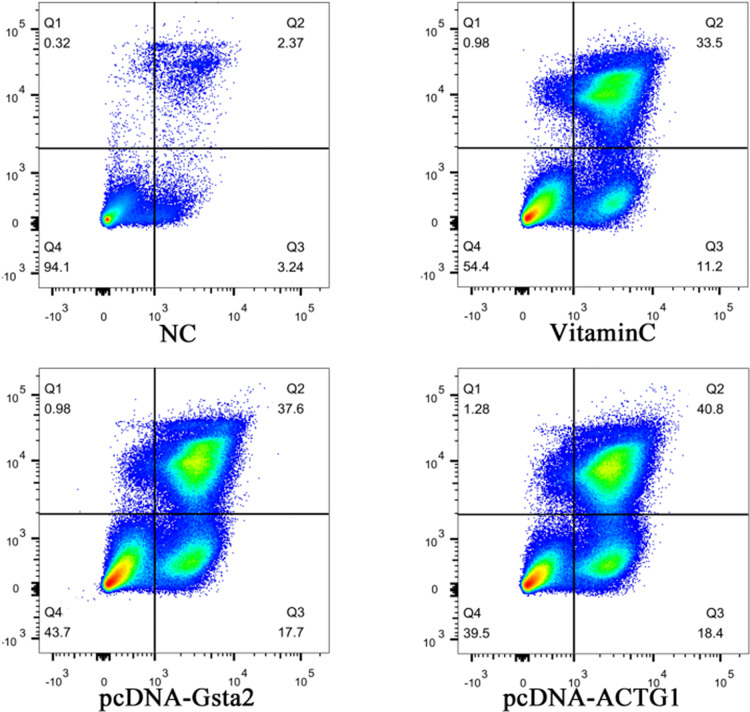
The survival rate of SH-SY5Y cells with overexpression of different genes by flow cytometry

**Figure 9:**
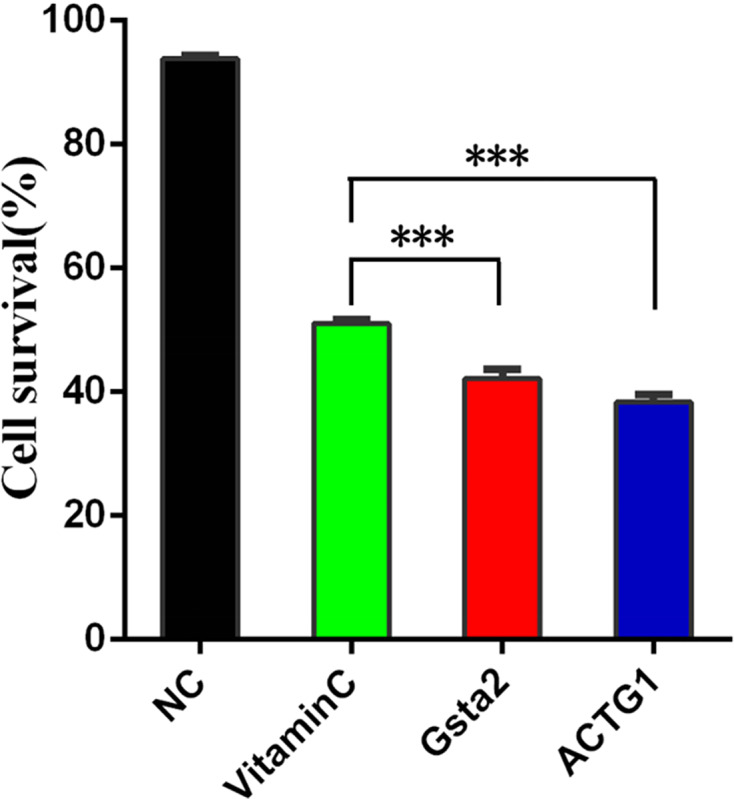
The histogram of survival rates in different groups measured by flow cytometry using SPSS16.0 software

We further overexpressed Gsta2 and Actg1 genes in PD model cells, and detected apoptosis with CCK-8 apoptosis kit and flow cytometry. The CCK-8 kit can directly detect cell apoptosis, whereas flow cytometry is used to detect the cell survival rate, to which the apoptosis rate is inversely proportional. The results showed a significant difference in apoptosis between cells overexpressing Gsta2 and the vitamin C control group (*P* = 0.0008, *P*<0.05) and between cells overexpressing Actg1 and the vitamin C control group (*P*<0.0001, *P*<0.05). Figure 7 shows the apoptosis results determined by the CCK-8 apoptosis kit; Figure 8 shows the SH-SY5Y cell survival rate determined by flow cytometry. Table 2 lists the numerical value of comparison that shows the cell survival rate by flow cytometry and Figure 9 the quantification and visualization of flow cytometry data using SPSS16.0 software.

**Table 2:** The numerical value of comparison which shows the cell survival rate in different groups by flow cytometry

Cell survival rate (%)	Exp 1	Exp 2	Exp 3
No-treatment control (NC)	93.2	94.1	94.2
Vitamin C	51.7	54.4	51.1
Gsta2	43.7	40.6	42.2
Actg1	39.5	37.1	38.4

## Discussion

In our previous research, capsaicin has been found to have neuroprotective effects in 6-OHDA-induced PD rats, and it has been found that capsaicin can lead to DA cell survival and behavioral recovery. A possible mechanism is through the effects on anti-oxidant stress and scavenging free radicals. Therefore, we wanted to verify whether capsaicin has a protective effect on a PD cell model *in vitro* and determine the possible molecular mechanism. We found that Gsta2 was up-regulated and Actg1 was down-regulated in microarray analysis of wax blocks containing the striatum from 6-OHDA-induced PD rats. We further constructed the overexpression vectors of the corresponding genes and overexpressed it in cell lines. PD model cells overexpressing Gsta2 or Actg1 showed significantly greater apoptosis than vitamin C-treated control cells. Gsta2 is a glutathione transferase gene involved in oxidative stress and free radical scavenging. Actg1 is an actin gene involved in the establishment of the cytoskeleton and autophagy pathway. Overexpression of both gene increased apoptosis, indicating that the imbalance between them may be one of the mechanisms of cell damage in PD. Capsaicin can protect the cells and reduce the rate of apoptosis by regulating them.

Cell death is a complex process that is carefully regulated. The role and regulatory network of apoptosis, as the first recognized programmed cell death program, have gradually become clear. At the same time, autophagy is also a conservative method of cell self-degradation. Autophagy is a process of degradation and reuse of damaged organelles and macromolecules through lysosomes and, at a basic level, is necessary to maintain cell homeostasis. Autophagy is involved in the pathophysiological process of anti-aging, differentiation and development, immunity and clearance of microorganisms, and tumor and other diseases [28]. Under starvation, hypoxia, drugs and other factors, a double-layer membrane will form around the cell components to be degraded, and then the partitioned membrane will gradually extend until the cytoplasmic component to be degraded will be completely sealed, forming an autophagosome. The autophagosome is transported to the lysosome through the cytoskeleton microtubule system, and the two fuse to form the autophagolysosome [29,30]. Finally, the contents of the autophagolysosome are degraded by lysosomal enzyme and utilized by the cell. In recent years, autophagy has been proven to regulate cell death together with apoptosis. In some cases, autophagy inhibits apoptosis, acting as a survival pathway of cells, but autophagy itself can also induce cell death or act together with apoptosis and induce cell death as a backup mechanism in the case of defective apoptosis. The two pathways are interrelated and regulated [29,30].

The actin filament is the dynamic basis of autophagy and its components actin and myosin regulate multiple stages of autophagy. The main components of the cytoskeleton, microtubules, actin filaments and phase-linked proteins, play an important role in autophagy. In mammalian eukaryotic cells, actin filaments, under the control of related proteins, are used as scaffolds for the expansion of autophagy precursors, resulting in actin comet trailing and dragging autophagy, thus ensuring effective fusion of autophagy and lysosome [31]. Actin filaments are closely related to autophagy as the main component of the cytoskeleton. Recent studies have shown that dynamic changes in actin filaments are key to autophagy formation and migration in mammalian cells [32,33]. Actin filaments provide an unobstructed fiber network for the transport of membrane structures to autophagosomes in different parts of the cell [33]. The mutation and / or deletion of myosin and actin and the role of the cytoskeleton in human disease are emerging domains of research. However, none of these studies have examined changes in autophagy due to abnormal cytoskeleton. The role of autophagy impairment in cytoskeleton-related diseases needs to be further clarified. Studying the exact role of cytoskeleton components in various stages of autophagy will contribute to the further understanding of the pathogenesis of autophagy-related diseases and the development of new and innovative drugs.

One of our target genes, Actg1, is a member of the cytoskeleton actin family, involved in cytoskeleton construction and the autophagy pathway [33]. Our analysis of differentially expressed genes between the capsaicin treatment group and the control group showed that capsaicin treatment could reduce the expression of Actg1, and we further overexpressed Actg1 in the PD cell model, and the apoptosis rate of PD cells increased after transfection. These results suggest capsaicin may affect the formation of the cytoskeleton by down-regulating Actg1, which is a member of the cytoskeleton actin gene family, and further influence the formation and migration of autophagy, thereby reducing the rate of apoptosis and ultimately achieving cell protection.

The second differentially expressed gene identified in our study was Gsta2. Gsta2 is a member of the GST superfamily, which encodes multifunctional enzymes important in the detoxification of electrophilic molecules, including carcinogens, mutagens, and several therapeutic drugs, by conjugation with glutathione. GSTs are a superfamily of phase II metabolizing enzymes that catalyze the detoxification of a large range of endogenous and exogenous toxic compounds, playing an important role in protecting cells against damage, through glutathione conjugation with electrophilic substances. The polymorphic variation in these enzymes that affects their activity seems to be related to individual susceptibility to various human diseases, including cancer. Of the GST superfamily, the alpha-class GSTs have commonly been described as one of the most versatile classes, as this class is responsible for the detoxification of compounds such as bilirubin, bile acids and penicillin, thyroid and steroid hormones, allowing the solubilization and storage of these compounds in the liver. Among the alpha class, Gsta1 and Gsta2 are the most widely expressed in human tissues. Gsta2 is one functional antioxidant response element (ARE) that plays a role in defense against oxidative stress. Recently, one study demonstrated that up-regulation of Gsta may serve as a compensatory mechanism against elevated oxidative stress, which accompanies obesity. On the other hand, down-regulating Gsta2 can worsen defense against oxidative stress. The current study showed that oxidative stress is one of the key mechanisms in the progression of PD [34].

In our previous studies, rats in the 6-OHDA group presented abnormalities of the antioxidant system, resulting in a significant decrease in the activities of antioxidant enzymes. And our previous study also demonstrated the antioxidative properties of capsaicin *in vivo*: the antioxidant system was effectively enhanced by capsaicin [3,35]. A previous study showed that capsaicin enhanced neuroprotection by up-regulating antioxidant enzymes *in vitro* [3]. Interestingly, using Affymetrix GeneChip Whole Transcript Expression Arrays, we found that Gsta2 was up-regulated in the substantia nigra of PD rats treated with capsaicin, suggesting that capsaicin may also enhance the body's ability to defend against oxidative stress by up-regulating Gsta2. Overexpression of Gsta2 significantly increased the apoptosis rate *in vitro*. The oxidative stress response and the antioxidant stress response are in dynamic equilibrium. If the level of oxidative stress reaction is too strong, the antioxidant stress reaction is insufficient, the dynamic equilibrium state will be broken, causing cell damage. In the present study, we found that capsaicin may regulate the antioxidant stress system by up-regulating Gsta2 and exert an antioxidant stress effect to achieve a neuroprotective effect. Excessive or inadequate antioxidant stress can cause cell damage.

Capsaicin is expected to become a new drug to regulate autophagy and oxidative stress and be used in clinical treatment of PD. Whether capsaicin can protect the nervous system in other ways, the optimal use of capsaicin, the effective dose of capsaicin and many other related issues need to be studied *in vivo* and *in vitro*.

## Conclusion

The present study showed that regulation of Actg1 and Gsta2 is the possible mechanism by which capsaicin alleviates apoptosis in a cell model of 6-OHDA-induced Parkinson's disease. We demonstrated that capsaicin could down-regulate Actg1 and up-regulate Gsta2 in PD rats, reduce apoptosis and protect cells by regulating the autophagy pathway and oxidative stress pathway. Actg1 and Gsta2 are promising therapeutic targets for alleviating the progression of PD.

## Abbreviations

Actg1: actin gamma 1
ARE: antioxidant response element
GST: glutathione S-transferase
Gsta2: glutathione S-transferase alpha 2
IF: immunofluorescence
PD: Parkinson's disease
TRPV1: transient receptor potential vanilloid subfamily member 1
WB: western blot

## References

[1] Gardaneh M., Gholami M. and Maghsoudi N. (2011) Synergy between glutathione peroxidase-1 and astrocytic growth factors suppresses free radical generation and protects dopaminergic neurons against 6-hydroxydopamine. Rejuv. Res. 14, 195–204 10.1089/rej.2010.1080 PMC3093024 21222532

[2] Kalia L.V. and Lang A.E. (2015) Parkinson's disease. Lancet 386, 896–912 10.1016/S0140-6736(14)61393-3 25904081

[3] Zhao Z.X., Wang J.F., Wang L.L., Yao X.M., Liu Y.L., Li Y. et al. (2017) Capsaicin protects against oxidative insults and alleviates behavioral deficits in rats with 6-OHDA-induced Parkinson's disease via activation of TRPV1. Neurochem. Res. 42, 3431–3438 10.1007/s11064-017-2388-4 28861768

[4] Ochi T., Takaishi Y., Kogure K. and Yamauti I. (2003) Antioxidant activity of a new capsaicin derivative from capsicum annuum. J. Nat. Prod. 66, 1094–1096 10.1021/np020465y 12932131

[5] Lee C.Y.J., Kim M., Yoon S.W. and Lee C.H. (2003) Short-term control of capsaicin on blood and oxidative stress of rats in vivo. Phytother. Res. 17, 454–458 10.1002/ptr.1172 12748978

[6] Gangabhagirathi R. and Joshi R. (2015) Antioxidant activity of capsaicin on radiation-induced oxidation of murine hepatic mitochondrial membrane preparation. Res. Rep. Biochem. 5, 163–171

[7] Kumar P., Chand S., Chandra P. and Maurya P.K. (2015) Influence of dietary capsaicin on redox status in red blood cells during human aging. Adv. Pharm. Bull. 5, 583–586 10.15171/apb.2015.078 26819932 PMC4729342

[8] Zhukova E.M. and Makarova O.P. (2002) Effect of capsaicin on dynamics of neutrophil functional activity in Wistar rat venous blood. Bull. Exp. Biol. Med. 134, 233–235 10.1023/A:1021491014764 12511989

[9] Reyes-Escogido M.D., Gonzalez-Mondragon E.G. and Vazquez-Tzompantzi E. (2011) Chemical and pharmacological aspects of capsaicin. Molecules 16, 1253–1270 10.3390/molecules16021253 21278678 PMC6259610

[10] Caterina M.J., Schumacher M.A., Tominaga M., Rosen T.A., Levine J.D. and Julius D. (1997) The capsaicin receptor: a heat-activated ion channel in the pain pathway. Nature 389, 816–824 10.1038/39807 9349813

[11] Baker K., Raemdonck K., Dekkak B., Snelgrove R.J., Ford J., Shala F. et al. (2016) Role of the ion channel, transient receptor potential cation channel subfamily V member 1 (TRPV1), in allergic asthma. Resp. Res. 17, 67 10.1186/s12931-016-0384-x PMC4890475 27255083

[12] Immke D.C. and Gavva N.R. (2006) The TRPV1 receptor and nociception. Semin. Cell Dev. Biol. 17, 582–591 10.1016/j.semcdb.2006.09.004 17196854

[13] Maiese K. (2014) “Tripping out” with the TRP superfamily and TRPV1 for novel neuroprotection. Curr. Neurovasc. Res. 11, 91–93 10.2174/1567202611666140328115757 24678639

[14] Starowicz K., Cristino L. and Di Marzo V. (2008) TRPV1 receptors in the central nervous system: potential for previously unforeseen therapeutic applications. Curr. Pharm. Design 14, 42–54 10.2174/138161208783330790 18220817

[15] Bunnell T.M. and Ervasti J.M. (2010) Delayed embryonic development and impaired cell growth and survival in Actg1 null mice. Cytoskeleton 67, 564–572 10.1002/cm.20467 20662086 PMC2989386

[16] Sun Q., Wang Y.L., Zhang Y.G., Liu F., Cheng X., Hou N. et al. (2007) Expression profiling reveals dysregulation of cellular cytoskeletal genes in HBx-induced hepatocarcinogenesis. Cancer Biol. Ther. 6, 668–674 10.4161/cbt.6.5.3955 17873514

[17] Kang K.W., Lee S.J. and Kim S.G. (2005) Molecular mechanism of Nrf2 activation by oxidative stress. Antioxid. Redox Sign. 7, 1664–1673 10.1089/ars.2005.7.1664 16356128

[18] Bousova I., Kostakova S., Matouskova P., Bartikova H., Szotakova B. and Skalova L. (2017) Monosodium glutamate-induced obesity changed the expression and activity of glutathione S-transferases in mouse heart and kidney. Pharmazie 72, 257–259 29441869 10.1691/ph.2017.6886

[19] Cheng B.X., Maffi S.K., Martinez A.A., Acosta Y.P.V., Morales L.D. and Roberts J.L. (2011) Insulin-like growth factor-I mediates neuroprotection in proteasome inhibition-induced cytotoxicity in SH-SY5Y cells. Mol. Cell. Neurosci. 47, 181–190 10.1016/j.mcn.2011.04.002 21545837 PMC3113659

[20] Datki Z., Juhasz A., Galfi M., Soos K., Papp R., Zadori D. et al. (2003) Method for measuring neurotoxicity of aggregating polypeptides with the MTT assay on differentiated neuroblastoma cells. Brain Res. Bull. 62, 223–229 10.1016/j.brainresbull.2003.09.011 14698355

[21] Cheung Y.T., Lau W.K.W., Yu M.S., Lai C.S.W., Yeung S.C., So K.F. et al. (2009) Effects of all-trans-retinoic acid on human SH-SY5Y neuroblastoma as in vitro model in neurotoxicity research. Neurotoxicology 30, 127–135 10.1016/j.neuro.2008.11.001 19056420

[22] Oyarce A.M. and Fleming P.J. (1991) Multiple forms of human dopamine beta-hydroxylase in SH-SY5Y neuroblastoma cells. Arch. Biochem. Biophys. 290, 503–510 10.1016/0003-9861(91)90573-2 1929417

[23] Presgraves S.P., Ahmed T., Borwege S. and Joyce J.N. (2004) Terminally differentiated SH-SY5Y cells provide a model system for studying neuroprotective effects of dopamine agonists. Neurotox. Res. 5, 579–598 10.1007/BF03033178 15111235

[24] Tian L.L., Zhou Z., Zhang Q., Sun Y.N., Li C.R., Cheng C.H. et al. (2007) Protective effect of (+/-) isoborneol against 6-OHDA-induced apoptosis in SH-SY5Y cells. Cell. Physiol. Biochem. 20, 1019–1032 10.1159/000110682 17975304

[25] Tiong C.X., Lu M. and Bian J.S. (2010) Protective effect of hydrogen sulphide against 6-OHDA-induced cell injury in SH-SY5Y cells involves PKC/PI3K/Akt pathway. Br. J. Pharmacol. 161, 467–480 10.1111/j.1476-5381.2010.00887.x 20735429 PMC2989596

[26] Lopes F.M., Schroder R., da Frota M.L.C., Zanotto A., Muller C.B., Pires A.S. et al. (2010) Comparison between proliferative and neuron-like SH-SY5Y cells as an in vitro model for Parkinson disease studies. Brain Res. 1337, 85–94 10.1016/j.brainres.2010.03.102 20380819

[27] Ju M.S., Lee P., Kim H.G., Lee K.Y., Hur J., Cho S.H. et al. (2010) Protective effects of standardized Thuja orientalis leaves against 6-hydroxydopamine-induced neurotoxicity in SH-SY5Y cells. Toxicol. in Vitro 24, 759–765 10.1016/j.tiv.2009.12.026 20040370

[28] Silva S.N., Azevedo A.P., Teixeira V., Pina J.E., Rueff J. and Gaspar J.F. (2009) The role of GSTA2 polymorphisms and haplotypes in breast cancer susceptibility: a case-control study in the Portuguese population. Oncol. Rep. 22, 593–598 19639209 10.3892/or_00000477

[29] Kast D.J. and Dominguez R. (2015) WHAMM links actin assembly via the Arp2/3 complex to autophagy. Autophagy 11, 1702–1704 10.1080/15548627.2015.1073434 26291929 PMC4590594

[30] Holland P. and Simonsen A. (2015) Actin shapes the autophagosome. Nat. Cell Biol. 17, 1094–1096 10.1038/ncb3224 26316454

[31] Zhong Z.Y., Sanchez-Lopez E. and Karin M. (2016) Autophagy, inflammation, and immunity: a troika governing cancer and its treatment. Cell 166, 288–298 10.1016/j.cell.2016.05.051 27419869 PMC4947210

[32] Wachter B., Schurger S., Rolinger J., von Ameln-Mayerhofer A., Berg D., Wagner H.J. et al. (2010) Effect of 6-hydroxydopamine (6-OHDA) on proliferation of glial cells in the rat cortex and striatum: evidence for de-differentiation of resident astrocytes. Cell Tissue Res. 342, 147–160 10.1007/s00441-010-1061-x 20976472

[33] Lambrechts A., Van Troys M. and Ampe C. (2004) The actin cytoskeleton in normal and pathological cell motility. Int. J. Biochem. Cell B 36, 1890–1909 10.1016/j.biocel.2004.01.024 15203104

[34] Perrin B.J. and Ervasti J.M. (2010) The actin gene family: function follows isoform. Cytoskeleton 67, 630–634 10.1002/cm.20475 20737541 PMC2949686

[35] Lee J.G., Yon J.M., Lin C., Jung Y., Jung K.Y. and Nam S.Y. (2012) Combined treatment with capsaicin and resveratrol enhances neuroprotection against glutamate-induced toxicity in mouse cerebral cortical neurons. Food Chem. Toxicol. 50, 3877–3885 10.1016/j.fct.2012.08.040 22943972

